# A Rapid Beam Pointing Determination and Beam-Pointing Error Analysis Method for a Geostationary Orbiting Microwave Radiometer Antenna in Consideration of Antenna Thermal Distortions

**DOI:** 10.3390/s21175943

**Published:** 2021-09-04

**Authors:** Hualong Hu, Xiaochong Tong, He Li

**Affiliations:** 1 School of Remote Sensing and Information Engineering, Wuhan University, Wuhan 430079, China; 2 Institute of Surveying and Mapping, Information Engineering University, Zhengzhou 450001, China; txchr@zxiat.org (X.T.); lihe_5115@zxiat.org (H.L.); 3 Zhengzhou Xinda Institute of Advanced Technology, Zhengzhou 450001, China

**Keywords:** geostationary orbiting (GEO), microwave radiometer, antenna beam pointing (ABP), ABP determination, beam-pointing error (BPE), image navigation and registration (INR), matrix optics (MO), thermal distortion error (TDE)

## Abstract

When observing the Earth’s radiation signal with a geostationary orbiting (GEO) mechanically scanned microwave radiometer, it is necessary to correct the antenna beam pointing (ABP) in real time for the deviation caused by thermal distortions of antenna reflectors with the help of the on-board Image Navigation and Registration (INR) system during scanning of the Earth. The traditional ABP determination and beam-pointing error (BPE) analysis method is based on the electromechanical coupling principle, which usurps time and computing resources and thus cannot meet the requirement for frequent real-time on-board INR operations needed by the GEO microwave radiometer. For this reason, matrix optics (MO), which is widely used in characterizing the optical path of the visible/infrared sensor, is extended to this study so that it can be applied to model the equivalent optical path of the microwave antenna with a much more complicated configuration. Based on the extended MO method, the ideal ABP determination model and the model for determining the actual ABP affected by reflector thermal distortions are deduced for China’s future GEO radiometer, and an MO-based BPE computing method, which establishes a direct connection between the reflector thermal distortion errors (TDEs) and the thermally induced BPE, is defined. To verify the overall performance of the extended MO method for rapid ABP determination, the outputs from the ideal ABP determination model were compared to calculations from GRASP 10.3 software. The experimental results show that the MO-based ABP determination model can achieve the same results as GRASP software with a significant advantage in computational efficiency (e.g., at the lowest frequency band of 54 GHz, our MO-based model yielded a 4,730,000 times faster computation time than the GRASP software). After validating the correctness of the extended MO method, the impacts of the reflector TDEs on the BPE were quantified on a case-by-case basis with the help of the defined BPE computing method, and those TDEs that had a significant impact on the BPE were therefore identified. The methods and results presented in this study are expected to set the basis for the further development of on-board INR systems to be used in China’s future GEO microwave radiometer and benefit the ABP determination and BEP analysis of other antenna configurations to a certain extent.

## 1. Introduction

The microwave radiometer is a passive remote sensing instrument which receives electromagnetic radiation from atmospheric, land, and oceanic environments through a reflector antenna to obtain the radiative properties of the viewed scene. Ever since the Microwave Sounding Unit (MSU) [1] on TIROS-N (1978) was first applied to the operational sounding of the Earth’s atmosphere, the space-borne microwave radiometer has been widely used for its advantages in weather forecasting, disaster early warning, and environmental monitoring [2,3,4]. However, constrained by the current state of the art in instruments and satellite platforms, all in-orbit microwave radiometers to date have been deployed in low-Earth orbit (LEO) with an altitude below 1000 km. To meet the requirement of a higher frequency of Earth observation for meteorological applications (e.g., operational weather nowcasting, short-term forecasting, and continuous monitoring of highly dynamic phenomena such as severe convective storms and tropical cyclones), the deployment of microwave radiometers in geostationary orbit (GEO) has gradually attracted worldwide attention. Technically, a GEO microwave radiometer can be realized using either a real- or a synthetic-aperture system. GeoSTAR [5,6] from the U.S., GAS [7] from the E.U., and GIMS [8,9,10], GIMS-II [11,12], as well as CSMIR [13] from China are representative of synthetic-aperture schemes. Because the synthetic-aperture system is inferior to the real-aperture system in terms of system complexity and calibration, scan flexibility, and real-time sounding of dynamic time-varying targets (e.g., clouds, rain, and atmosphere), none of the above projects have entered the space-borne stage. The real-aperture schemes are represented by GEM [14] from the U.S., GOMAS [15,16,17] from the E.U., and GeoMWR [18] from China. However, due to technical difficulties in the large-aperture antenna, such as processing and manufacturing, assembly and unfolding, mechanical scanning, radiometric calibration, and on-board thermal distortion compensation, the above projects also remain in the scheme design stage.

Owing to the limited instantaneous field of view (IFOV) of the antenna beam, the GEO microwave radiometer requires the commanded two-dimensional (2-D) attitude maneuvers of an agile satellite to help implement beam coverage in the particular scene. Ideally, if the satellite could maneuver properly in an ideal geostationary orbit in accordance with the pre-planned scan instructions, and the antenna of the microwave radiometer was free of thermal distortions, the radiometer would provide the end user with near-real-time radiometric data that were perfectly preregistered with respect to a nominal fixed grid valid for the GEO satellite [19,20]. In this case, the sampled radiometric observations could be directly transmitted to the follow-on GEO meteorological applications (without the time-consuming and laborious postprocessing of re-geolocation) to ensure timeliness. However, in practice, influenced by many disturbing factors (e.g., platform vibration, orbital motion, attitude deviation, solar and lunar gravity, and orbital heat flux), the mounting positions and mounting angles of antenna elements will inevitably change, thus making the actual antenna beam pointing (ABP) deviate from the ideal settings. This would cause the acquired samples to drift away from their corresponding nominal grid cells and consequently affect the accuracy of their subsequent applications. The development of modern numerical weather prediction (NWP) technology proposes higher requirements for the direct geolocation accuracy of satellite radiometer data. For GEO microwave radiometers in particular, a slight deviation in the ABP will have a significant impact on the absolute positioning accuracy of the radiometric data. When the absolute positioning accuracy at the GEO sub-satellite point (SSP) is in the magnitude of $1\times 10^{3}\;m$, the ABP accuracy should be better than 28 μrad (about $6^{\prime \prime }$). For the GEO three-axis stabilized satellite platform, its high orbital height (about 36,000 km) and periodic entry and exit into the Earth’s shadow cause antenna reflectors exposed outside the spacecraft to undergo intense alternating heating and cooling by the sun and the cold universe. Therefore, among the sources of disturbances that cause the beam-pointing error (BPE), structural thermal distortions of the antenna reflectors due to the thermal environment around the GEO satellite have the most significant impact on the BPE [21]. To achieve the ABP accuracy required by the GEO microwave radiometer, it is necessary to introduce the Image Navigation and Registration (INR) system [22] that was first applied to the GEO optical meteorological satellites to compensate for the BPE caused by the perturbing effects described previously (especially for the effect corresponding to antenna thermal distortions) while the radiometer is scanning the desired Earth region.

From the viewpoint of the INR system, a rapid and accurate method to calculate the ABP under ideal and thermally disturbing conditions and assess the relationship between numerous antenna thermal distortion errors (TDEs) and the associated thermally induced BPE is highly desired. Currently, the most used methodology to study the antenna thermal distortion effects on BPE is the electromechanical coupling principle [23,24,25,26]. First, the thermo-structural finite element model is used to simulate the thermally deformed antenna in a given temperature field. Then, a “geometric-electromagnetic” model is established in electromagnetic field simulation software (such as FEKO, HFSS, and GRASP), and the three-dimensional (3-D) far-field antenna pattern and other antenna performance indicators (including the ABP) at the given temperature are calculated using a physical optics (PO) approximation. Theoretically, the electromechanical coupling principle is a rigorous methodology to study the thermal distortion’s effects on antenna performance. This approach allows not only an accurate calculation of ABP but also gives the relationship between thermal distortions and other antenna performance indicators (e.g., antenna gain, main lobe width, sidelobe level, beam efficiency, and co- and cross-polarization levels) [26]. However, in the process of in-orbit operation, the electromechanical coupling methodology cannot be performed rapidly enough to maintain pace with the routine INR operations due to its high computational complexity. Therefore, how to accurately describe the beam propagation geometry inside the antenna with or without the presence of antenna reflector thermal distortion effects and analyze the contribution of each thermal distortion on the direction of propagation of the antenna beam has become an urgent fundamental issue that needs to be solved before the GEO microwave radiometer is put into practical application. At the time of this paper, the research on this issue has not been addressed yet; thus, a focused discussion of this issue remains necessary.

In this study, by taking China’s future GEO microwave radiometer as an example, the rapid ABP determination and BPE analysis method suitable for the mechanically scanned microwave antenna are studied, considering the thermal distortion effects of antenna reflectors. The contributions of this study mainly lie in three aspects:

(1) The method of matrix optics (MO), which is widely used in characterizing the optical path of the visible/infrared sensor, is adapted for modeling the equivalent optical path of the microwave antenna with a much more complicated configuration.
(2) The ideal ABP determination model and the model for determining the actual ABP affected by thermal distortions of the antenna reflectors are deduced for the examined GEO microwave antenna. Compared with the PO approximation, the primary advantage of the extended MO method is that both the ideal and actual ABPs can be rapidly and accurately computed with simple matrix operations, thus effectively reducing the computational burden of the onboard computer.
(3) Based on the ideal and actual ABP determination models, the MO-based method for calculating the thermally induced BPE is given. The significance of the MO-based BPE computing method lies in its capability to establish a direct connection between reflector thermal distortions and the resulting BPE. Through numerical experiments, the impacts of different reflector thermal distortions on the BPE are quantified on a case-by-case basis, and those thermal distortions that have a significant impact on the BPE are identified.

The methods and results in this study set the basis for the further development of an onboard INR system that will be used in China’s future GEO microwave radiometer and build a paradigm for the real-time ABP determination and BPE analysis of microwave antennas with different configurations.

## 2. Instrument Overview

### 2.1. Geometric Structure of the Microwave Antenna

**Figure 1:**
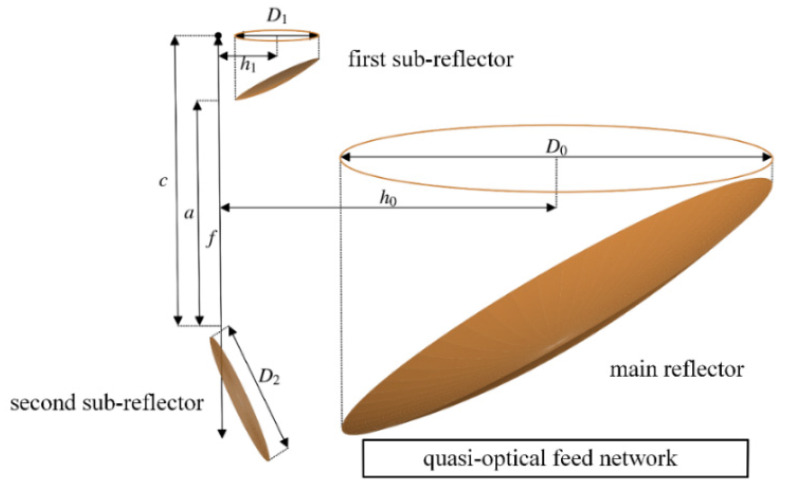
The geometry of the GEO microwave antenna that was used in this study.

The subject, a GEO microwave radiometer (i.e., GeoMWR in [18]), adopts the classical offset Cassegrain reflector antenna attached with a planar folding mirror (Figure 1). It is mainly composed of a reflector system and a multi-band quasi-optical feed (QOF) system.

The reflector system consists of three reflectors (i.e., a main reflector (MR), a first sub-reflector (FSR), and a second sub-reflector (SSR)). The MR is an offset parabolic reflector (f is the focal length of the parent paraboloid), which is cut from one side of the rotationally symmetric parabolic reflector by a cylinder with an aperture of $D_{0}$ and an offset height of $h_{0}$. The FSR is an offset hyperbolic reflector (2a and 2c are the major axis length and inter-focal distance of the parent hyperboloid, respectively), which is cut from the concave hyperboloid by a cylinder with an aperture of $D_{1}$ and an offset height of $h_{1}$. The aperture of the FSR is much smaller than that of the MR (i.e., $D_{1}\ll D_{0}$), and its virtual focus and focal axis coincide with those of the MR. The MR and the FSR form the classical offset Cassegrain antenna. The SSR is a planar folding mirror ($D_{2}$ is the aperture of the SSR), which is used to fold the beam propagation path to achieve a compact system layout.

The QOF system consists of a lightweight moving part, frequency-selective surfaces, wire grid polarizers, a series of planar, hyperbolic, and ellipsoidal mirrors, and corrugated feed horns used at different frequency bands. The lightweight moving part makes a rapid circular rotation around its rotating shaft, generating conical scanning of the antenna beam. After the frequency and polarization separations by the frequency-selective surfaces and wire grid polarizers, the incoming broadband signal beam is split into separate beams and transmitted by a series of planar, hyperbolic, and ellipsoidal mirrors to the input feed horns working at different frequency bands [18,27]. Compared with the traditional multi-beam focal plane array of the horns, the QOF system can receive and transmit multiple frequency bands simultaneously and therefore allow the electrical boresights of different frequency bands to be coaxially aligned on a common boresight [28].

### 2.2. Operational Principle of the Microwave Antenna

**Figure 2:**
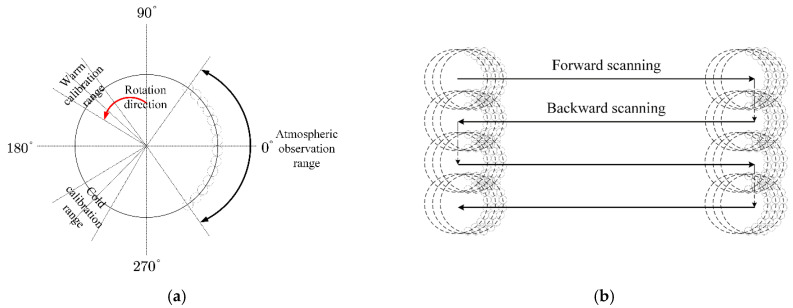
The operational principle of the GEO microwave antenna in this study. (**a**) 1-D LFS. (**b**) 2-D OSS.

The operational principle of the GeoMWR is shown in Figure 2. Since the GeoMWR is designed to operate in GEO (where the relative motion of the Earth and the satellite are nulled), it requires a two-dimensional (2-D) beam scanning mechanism to realize the narrow-beam coverage in a regional viewed scene. The GeoMWR implements 2-D beam scanning in a combination of 1-D local fast scanning (LFS) and 2-D overall slow scanning (OSS) [18]. In the process of 1-D LFS, the moving part in the QOF system rotates at a high speed around its rotating shaft under the control of the servo-drive system, driving the antenna beam to conduct conical scanning around the cone axis that is parallel to the focal axis of the MR. Throughout the entire scan cycle, the antenna beam receives the radiation from the viewed scene, the hot calibration source, and the cold cosmic background in turn (Figure 2a). Along with the 1-D LFS, the satellite platform performs 2-D OSS along the east–west and north–south directions. The GEO satellite scans the antenna beam in the east–west direction to produce an east–west longitudinal swath. At the end of a swath, the GEO satellite steps in the north–south direction to a new scan swath (Figure 2b) [29]. Since ABP determination outside the antenna is much simpler than inside the antenna, the 2-D OSS effect on the ABP is not considered in developing the ABP determination model.

**Figure 3:**
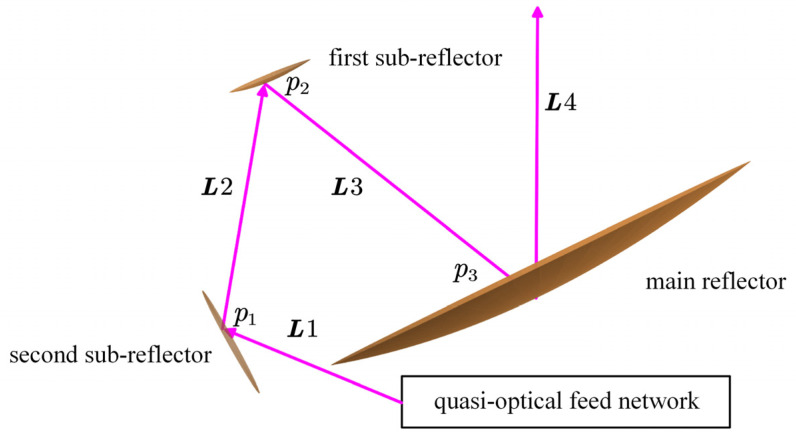
The reverse propagation path of the beam within the GEO microwave antenna.

In the real case, the broadband antenna beam transmitted from the viewed scene is first reflected from the MR to the FSR. From the FSR, the antenna beam is reflected again to the SSR and finally enters the QOF system, where it is divided into separate beams and received by the feed horns working at different frequency bands. To facilitate the development of the ABP determination model, we assume that the propagation direction of the antenna beam is reversed: the antenna beam is emitted from the QOF system (path L1) and is reflected off the SSR (path L2), the FSR (path L3), and the MR (path L4) in turn (as shown in Figure 3, where the thick purple lines represent the pointing vectors of the beam (i.e., the maximum gain direction)). When the moving part in the QOF system is rotated, the emergent beam generates a cone-shaped scan pattern, as previously discussed.

## 3. ABP Determination Models and BPE Computing Method

The ABP determination model discussed in this study describes the beam propagation geometry within the antenna. The pointing information of the beam emerging from the MR, which is specified with respect to the instrument coordinate system (ICS), can be obtained from the model with a series of matrix operations. The term “ideal” ABP used in this study implies that the antenna is not thermally deformed (i.e., every antenna element maintains the ideal position and attitude (P&A) desired by the users). In contrast, the term “actual” ABP implies that each reflector of the antenna subjected to temperature fluctuations undergoes an overall motion (i.e., the true P&A values of each reflector deviate from the nominal values). Based on extending the traditional MO method, this section deduces the ideal and actual ABP determination models and then gives an MO-based method for fast calculation of the thermally induced BPE.

### 3.1. Definition of Basic Coordinate Systems

**Figure 4:**
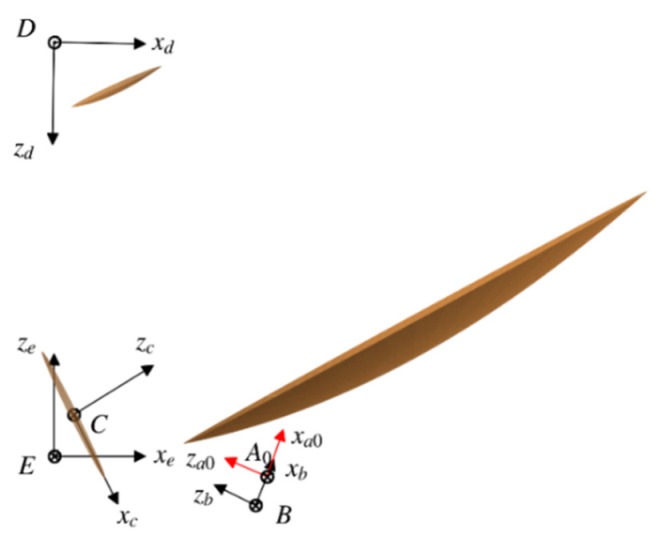
Definition of basic BFCSs that are used in deducing the ideal ABP determination model.

This part defines the ideal body-fixed coordinate system (BFCS) for each antenna element. It should be pointed out that the QOF system is a black box system for users other than the instrument manufacturer, and the beam transmission process in it is confidential. Therefore, we equate the rotation of the lightweight moving part in the QOF system to the circular motion of an equivalent coaxial feed (ECF). The fixed rotation axis around which the ECF is rotated is called the feed rotation axis (FRA). According to Figure 4, the definitions for five basic BFCSs are given:

(1) ECF coordinate system A-$x_{a}y_{a}z_{a}$

The origin A of the coordinates is placed at the geometric centroid of the ECF, and the $z_{a}$-axis coincides with the outward-pointing normal at the entrance of the ECF. Ideally, the antenna beam is assumed to enter the ECF along the negative $z_{a}$-axis. Particularly, when the ECF is located at the “zero” position, the corresponding ECF coordinate system is denoted as A0-$x_{a0}y_{a0}z_{a0}$. The ICS coordinates of the origin A0 are $\left(x_{A0},y_{A0},z_{A0}\right)$, and the virtual mounting matrix of the “zero-position” ECF is $R_{\mathrm{ICS}}^{\mathrm{ECF}0}=R_{z}\left(\alpha_{a0}\right)R_{y}\left(\beta_{a0}\right)R_{z}\left(\gamma_{a0}\right)$.

(2) FRA coordinate system B-$x_{b}y_{b}z_{b}$

The FRA coordinate system is an auxiliary coordinate system established to describe the circular rotation of the ECF, where the $z_{b}$-axis is specified as the rotation axis of the ECF. The ICS coordinates of the origin B are $\left(x_{B},y_{B},z_{B}\right)$, and the virtual mounting matrix of the FRA is $R_{\mathrm{ICS}}^{\mathrm{FRA}}=R_{z}\left(\alpha_{b}\right)R_{y}\left(\beta_{b}\right)R_{z}\left(\gamma_{b}\right)$.

(3) SSR coordinate system C-$x_{c}y_{c}z_{c}$

The origin C of the coordinates is placed at the geometric centroid of the SSR, and the $z_{c}$-axis is specified as the outward-pointing normal of the SSR. The ICS coordinates of the origin C are $\left(x_{C},y_{C},z_{C}\right)$, and the mounting matrix of the SSR is $R_{\mathrm{ICS}}^{\mathrm{SSR}}=R_{z}\left(\alpha_{c}\right)R_{y}\left(\beta_{c}\right)R_{z}\left(\gamma_{c}\right)$.

(4) FSR coordinate system D-$x_{d}y_{d}z_{d}$

The origin D of the coordinates is placed at the virtual focus of the FSR, and the $z_{d}$-axis is specified as the focal axis of the FSR. The ICS coordinates of the origin D are $\left(x_{D},y_{D},z_{D}\right)$, and the mounting matrix of the FSR is $R_{\mathrm{ICS}}^{\mathrm{FSR}}=R_{z}\left(\alpha_{d}\right)R_{y}\left(\beta_{d}\right)R_{z}\left(\gamma_{d}\right)$.

(5) MR coordinate system E-$x_{e}y_{e}z_{e}$

The origin E of the coordinates is placed at the vertex of the parent paraboloid of the MR, and the $z_{e}$-axis is specified as the focal axis of the MR. The MR is mounted on the Earth-facing panel of the GEO satellite, and its focal axis points to the Earth. It should be noted that the MR coordinate system is also taken as the ICS.

### 3.2. Ideal ABP Determination Model

For optical sensors, the traditional MO method is used to determine the pointing vector (PV) of each image pixel in the ICS [20,30]. The key aspect of this method is to use the 3-by-3 reflection matrix (RM) of each optical mirror to describe the propagation path of the optical ray from the ground location to the corresponding pixel location in the ICS. Since we were only concerned with the pointing information of the antenna beam rather than other antenna performance indicators, it was reasonable to adopt the MO method for rapid ABP determination.

However, it must be pointed out that the ABP determination is quite different from the pointing determination of the optical ray. Compared with optical sensors, space-borne microwave antennas include not only planar reflectors but also curved reflectors. For a planar reflector, the outward-pointing normal direction is the same for arbitrary points on the surface. Thus, the translation of the incident beam on the planar reflector does not affect the pointing of the reflected beam, while for a curved reflector, the outward-pointing normals are in different directions at different points on the surface. As a result, a small drift in the point of emergence (POE) of the beam will result in a relatively large deviation in the pointing of the reflected beam. Therefore, the key point to developing the ABP determination model is to trace the beam’s PV and POE simultaneously as it propagates in the reflector system.

To describe the propagation of the antenna beam in a more unified and compact form, we extended the traditional 3-D PV to a 7-D generalized pointing vector (GPV):

(1) $$L={\left[\begin{array}{lll} l & r & 1 \end{array}\right]}^{T}={\left[\begin{array}{lllllll} l_{x} & l_{y} & l_{z} & r_{x} & r_{y} & r_{z} & 1 \end{array}\right]}^{T}$$

where $l={\left[\begin{array}{lll} l_{x} & l_{y} & l_{z} \end{array}\right]}^{T}$ is the traditional 3-D PV and $r={\left[\begin{array}{lll} r_{x} & r_{y} & r_{z} \end{array}\right]}^{T}$ represents the coordinates of the POE of the beam in a specific coordinate system.

To be compatible with the 7-D GPV, we further extended the traditional 3-by-3 attitude transformation matrix (ATM) and RM to a 7-by-7 generalized transformation matrix (GTM) $M_{i}^{j}$ and generalized reflection matrix (GRM) $F_{i}$, respectively. The explicit expressions for $M_{i}^{j}$ and $F_{i}$ are as follows:

(2) $$M_{i}^{j}=\left[\begin{array}{lll} Rot_{i}^{j} & O_{3\times 3} & O_{3\times 1}\\ O_{3\times 3} & Rot_{i}^{j} & I_{j}\\ O_{1\times 3} & O_{1\times 3} & 1 \end{array}\right]$$

(3) $$\{\begin{array}{l} F_{i}=\left[\begin{array}{lll} Ref_{i} & O_{3\times 3} & O_{3\times 1}\\ k_{i}E_{3\times 3} & E_{3\times 3} & O_{3\times 1}\\ O_{1\times 3} & O_{1\times 3} & 1 \end{array}\right]\\ Ref_{i}=\left[\begin{array}{lll} 1-2n_{x}^{2} & -2n_{x}n_{y} & -2n_{x}n_{z}\\ -2n_{x}n_{y} & 1-2n_{y}^{2} & -2n_{y}n_{z}\\ 2n_{x}n_{z} & -2n_{y}n_{z} & 1-2n_{z}^{2} \end{array}\right] \end{array}$$

where $Rot_{i}^{j}$ is the 3-by-3 ATM which transforms the pointing vectors between representations from coordinate system i (whose origin is denoted as I) to coordinate system j (whose origin is denoted as J), $I_{j}$ is the j coordinate representation of the origin I, $Ref_{i}$ is the 3-by-3 RM at a certain point in coordinate system i, ${\left[\begin{array}{lll} n_{x} & n_{y} & n_{z} \end{array}\right]}^{T}\;{\left[\begin{array}{lll} n_{x} & n_{y} & n_{z} \end{array}\right]}^{T}$ is the outward-pointing normal to a reflector at this particular point; $k_{i}$ is the optical path length of the incident beam, and O and E represent the zero matrix (vector) and identity matrix, respectively.

On the basis of the above definitions, now, we can deduce the ideal ABP determination model. Referring to Figure 3, let us denote the ECF coordinate representation of the GPV of the ECF-emitted beam L1 as $L1_{\mathrm{ECF},\mathrm{out}}$. According to Section 2.2, the ECF-emitted beam emerges along the positive $z_{a}$-axis from the origin A of the ECF coordinate system. Thus, $L1_{\mathrm{ECF},\mathrm{out}}$ can be represented in the following form:

(4) $$L1_{\mathrm{ECF},\mathrm{out}}={\left[\begin{array}{lllllll} 0 & 0 & 1 & 0 & 0 & 0 & 1 \end{array}\right]}^{T}$$

In the process of 1-D LFS, we assume the initial scan azimuth of the ECF is $\phi_{q0}$ ($\phi_{q0}$ is measured counterclockwise from the positive $x_{a}$-axis), the angular velocity of the ECF is ω, and the initial scanning time of the current scan swath is $t_{0}$. Therefore, for any moment t, the FRA coordinate representation of the GPV L1 can be obtained as follows:

(5) $$\{\begin{array}{l} L1_{\mathrm{FRA},\mathrm{out}}=M_{\mathrm{ECF}}^{\mathrm{FRA}}\left(t\right)L1_{\mathrm{ECF},\mathrm{out}}\\ M_{\mathrm{ECF}}^{\mathrm{FRA}}\left(t\right)=\left[\begin{array}{ccc} Rot_{z}\left(t\right)Rot_{\mathrm{ECF}0}^{\mathrm{FRA}} & O_{3\times 3} & O_{3\times 1}\\ O_{3\times 3} & Rot_{z}\left(t\right)Rot_{\mathrm{ECF}0}^{\mathrm{FRA}} & A_{\mathrm{FRA}}\left(t\right)\\ O_{1\times 3} & O_{1\times 3} & 1 \end{array}\right] \end{array}$$

where $Rot_{\mathrm{ECF}0}^{\mathrm{FRA}}$ represents the 3-by-3 ATM from the “zero-position” ECF coordinate system to the FRA coordinate system, $Rot_{z}\left(t\right)$ represents the vector rotation matrix rotating the “zero-position” ECF through an angle of $\left(\phi_{q0}+\omega \left(t-t_{0}\right)\right)$ around the $z_{b}$-axis of the FRA coordinate system, the angle of $\left(\phi_{q0}+\omega \left(t-t_{0}\right)\right)$ is the scan azimuth of the ECF at moment t, and $A_{\mathrm{FRA}}\left(t\right)$ is the FRA coordinate representation of the origin A of the ECF system. Here, $Rot_{z}\left(t\right)$ and $A_{\mathrm{FRA}}\left(t\right)$ can be explicitly computed as follows:

(6) $$\{\begin{array}{l} Rot_{z}\left(t\right)=\left[\begin{array}{lll} c\left(t\right) & -s\left(t\right) & 0\\ s\left(t\right) & c\left(t\right) & 0\\ 0 & 0 & 1 \end{array}\right]\\ A_{\mathrm{FRA}}\left(t\right)=Rot_{z}\left(t\right)Rot_{\mathrm{ICS}}^{\mathrm{FRA}}{\left[\begin{array}{l} x_{A0}-x_{B}\\ y_{A0}-y_{B}\\ z_{A0}-z_{B} \end{array}\right]}_{\mathrm{ICS}} \end{array}$$

where $c\left(t\right)=\cos\left(\phi_{q0}+\omega \left(t-t_{0}\right)\right)$ and $s\left(t\right)=\sin\left(\phi_{q0}+\omega \left(t-t_{0}\right)\right)$.

To obtain the GPV $L2_{\mathrm{SSR},\mathrm{out}}$ representing the beam reflected off the SSR, we can first transform the ECF-emitted beam $L1_{\mathrm{FRA},\mathrm{out}}$ defined in the FRA coordinate system into the incident beam $L1_{\mathrm{SSR},\mathrm{in}}$ defined in the SSR coordinate system. Using a similar calculation process to that shown above, $L1_{\mathrm{SSR},\mathrm{in}}$ can be obtained as follows:

(7) $$\{\begin{array}{l} L1_{\mathrm{SSR},\mathrm{in}}=M_{\mathrm{FRA}}^{\mathrm{SSR}}L1_{\mathrm{FRA},\mathrm{out}}\\ M_{\mathrm{FRA}}^{\mathrm{SSR}}=\left[\begin{array}{lll} Rot_{\mathrm{FRA}}^{\mathrm{SSR}} & O_{3\times 3} & O_{3\times 1}\\ O_{3\times 3} & Rot_{\mathrm{FRA}}^{\mathrm{SSR}} & B_{\mathrm{SSR}}\\ O_{1\times 3} & O_{1\times 3} & 1 \end{array}\right] \end{array}$$

where $Rot_{\mathrm{FRA}}^{\mathrm{SSR}}$ is the 3-by-3 ATM from the FRA coordinate system to the SSR coordinate system and $B_{\mathrm{SSR}}$ is the SSR coordinate representation of the origin B of the FRA coordinate system.

Assuming that the point of incidence (POI) where the incident beam $L1_{\mathrm{SSR},\mathrm{in}}$ falls on the SSR is $p_{1}$, and the coordinates of $p_{1}$ in the SSR coordinate system are $p_{1,\mathrm{SSR}}$, according to the incidence geometry, $p_{1,\mathrm{SSR}}$ takes the following form:

(8) $${\left[\begin{array}{l} p_{1,x}\\ p_{1,y}\\ p_{1,z} \end{array}\right]}_{\mathrm{SSR}}={\left[\begin{array}{l} r1_{x}\\ r1_{y}\\ r1_{z} \end{array}\right]}_{\mathrm{SSR},\mathrm{in}}+k_{\mathrm{SSR},\mathrm{in}}{\left[\begin{array}{l} l1_{x}\\ l1_{y}\\ l1_{z} \end{array}\right]}_{\mathrm{SSR},\mathrm{in}}$$

where $r1_{\mathrm{SSR},\mathrm{in}}$ and $l1_{\mathrm{SSR},\mathrm{in}}$ are the POE component and PV component of $L1_{\mathrm{SSR},\mathrm{in}}$ and $k_{\mathrm{SSR},\mathrm{in}}$ is the optical path length from point A to point $p_{1}$. Considering that the equation of the SSR, which is defined in the SSR coordinate system, is z=0, thus, by solving the pair of simultaneous equations z=0 and Equation (8), $k_{\mathrm{SSR},\mathrm{in}}$ can be obtained:

(9) $$k_{\mathrm{SSR},\mathrm{in}}={\left(-r1_{z}/l1_{z}\right)}_{\mathrm{SSR},\mathrm{in}}$$

As noted before, the outward-pointing normals at different points on the SSR are always the same (while for the curved reflectors (i.e., the FSR and MR), the outward-pointing normal should be computed by taking the first-order partial derivatives of the implicitly defined reflector at the POI); thus, the outward-pointing normal at POI $p_{1}$ can be directly written as follows:

(10) $$n_{\mathrm{SSR}}\left(p_{1}\right)={\left[\begin{array}{lll} n_{\mathrm{SSR},x}\left(p_{1}\right) & n_{\mathrm{SSR},y}\left(p_{1}\right) & n_{\mathrm{SSR},z}\left(p_{1}\right) \end{array}\right]}^{T}={\left[\begin{array}{lll} 0 & 0 & 1 \end{array}\right]}^{T}$$

Then, the GRM $F_{\mathrm{SSR}}\left(p_{1}\right)$ of the SSR at the POI $p_{1}$ can be achieved by substituting $k_{\mathrm{SSR},\mathrm{in}}$ and $n_{\mathrm{SSR}}\left(p_{1}\right)$ into Equation (3). According to the above calculations, $L2_{\mathrm{SSR},\mathrm{out}}$ can be calculated as follows:

(11) $$L2_{\mathrm{SSR},\mathrm{out}}=F_{\mathrm{SSR}}\left(p_{1}\right)L1_{\mathrm{SSR},\mathrm{in}}$$

So far, we have used the extended MO method to accomplish the calculation of $L2_{\mathrm{SSR},\mathrm{out}}$, representing the beam reflected off the SSR. By repeating similar steps, $L3_{\mathrm{FSR},\mathrm{out}}$ (the GPV of the beam reflected off the FSR) and $L4_{\mathrm{MR},\mathrm{out}}$ (the GPV of the beam reflected off the MR) can be obtained. The detailed calculation process for $L3_{\mathrm{FSR},\mathrm{out}}$ and $L4_{\mathrm{MR},\mathrm{out}}$ will not be repeated here.

For ease of description, let us denote the GTM from the SSR coordinate system to the FSR coordinate system as $M_{\mathrm{SSR}}^{\mathrm{FSR}}$, the GTM from the FSR coordinate system to the MR coordinate system as $M_{\mathrm{FSR}}^{\mathrm{MR}}$, the GRM of the FSR at the POI $p_{2}$ as $F_{\mathrm{FSR}}\left(p_{2}\right)$, and the GRM of the MR at the POI $p_{3}$ as $F_{\mathrm{MR}}\left(p_{3}\right)$. Then, the ideal ABP determination model can be expressed as

(12) $$\{\begin{array}{l} L_{\mathrm{ICS},\mathrm{out}}\left(t\right)=F_{\mathrm{MR}}\left(p_{3}\right)M_{\mathrm{FSR}}^{\mathrm{MR}}F_{\mathrm{FSR}}\left(p_{2}\right)M_{\mathrm{SSR}}^{\mathrm{FSR}}F_{\mathrm{SSR}}\left(p_{1}\right)M_{\mathrm{FRA}}^{\mathrm{SSR}}M_{\mathrm{ECF}}^{\mathrm{FRA}}\left(t\right)L1_{\mathrm{ECF},\mathrm{out}}\\ L1_{\mathrm{ECF},\mathrm{out}}={\left[\begin{array}{lllllll} 0 & 0 & 1 & 0 & 0 & 0 & 1 \end{array}\right]}^{T} \end{array}\cdot$$

### 3.3. Definition of the Thermal Distortion Errors of the Reflector

The ABP determination model given in Section 3.2 was deduced on the assumption that all antenna elements remained at the ideal P&A values. However, in practice, the actual ABP will deviate from the ideal ABP due to the thermal distortion effects of the antenna reflectors exposed outside the spacecraft. Before developing the actual ABP determination model, the necessary first step is to define the TDEs of the reflector.

In this study, only the rigid motion of the ideal reflector is considered. Thus, the reflector’s TDEs are defined as a result of the rigid motion of the actual reflector with respect to the ideal reflector. Specifically, the TDEs of the reflector include the translation error of the origin of the actual reflector’s BFCS relative to the origin of the ideal reflector’s BFCS, and the misalignment errors of each axis of the actual reflector’s BFCS are relative to the corresponding axis of the ideal reflector’s BFCS.

The transformation from the ideal reflector’s BFCS to the actual reflector’s BFCS can be reduced to a translation followed by a rotation. The origin of the ideal reflector’s BFCS first translates in the direction of $\left(x_{i,ma},y_{i,ma},z_{i,ma}\right)$, and then the translated result rotates in a 3-1-2 convention with three Euler angles $\left(\psi_{i,ma},\varphi_{i,ma},\theta_{i,ma}\right)$ (as measured counterclockwise from the positive z -, x -, and y-axes) to arrive at the actual reflector’s BFCS (note: subscript i={SSR, FRS, MR} is used to distinguish the TDEs of the SSR, FSR, and MR). Finally, for ease of description, we define the x-, y-, and z-axes of the reflector’s BFCS as the roll, pitch, and yaw axes, respectively. Therefore, $\varphi_{i,ma}$, $\theta_{i,ma}$, and $\psi_{i,ma}$ are called the roll, pitch, and yaw misalignment angles, and $x_{i,ma}$, $y_{i,ma}$, and $z_{i,ma}$ are called the roll, pitch, and yaw axial translations.

Based on the above definitions, the 7-by-7 GTM $M_{i^{\prime }}^{j^{\prime }}$ which transforms the pointing vectors between representations from the actual BFCS $i^{\prime }$ (whose origin is denoted as $I^{\prime }$) to the actual BFCS $j^{\prime }$ (whose origin is denoted as $J^{\prime }$), can be written in the following form:

(13) $$\{\begin{array}{l} M_{i^{\prime }}^{j^{\prime }}=\left[\begin{array}{lll} Rot_{i^{\prime }}^{j^{\prime }} & O_{3\times 3} & O_{3\times 1}\\ O_{3\times 3} & Rot_{i^{\prime }}^{j^{\prime }} & {I^{\prime }}_{j^{\prime }}\\ O_{1\times 3} & O_{1\times 3} & 1 \end{array}\right]\\ Rot_{i^{\prime }}^{j^{\prime }}=Rot_{j}^{j^{\prime }}\left(\varphi_{j,ma},\theta_{j,ma},\psi_{j,ma}\right)Rot_{i}^{j}{\left(Rot_{i}^{i^{\prime }}\left(\varphi_{i,ma},\theta_{i,ma},\psi_{i,ma}\right)\right)}^{T}\\ {I^{\prime }}_{j^{\prime }}=Rot_{j}^{j^{\prime }}\left(\varphi_{j,ma},\theta_{j,ma},\psi_{j,ma}\right)\left(-{J^{\prime }}_{j}+I_{j}+Rot_{i}^{j}{I^{\prime }}_{i}\right) \end{array}$$

where $Rot_{i^{\prime }}^{j^{\prime }}$ is the 3-by-3 ATM from the actual BFCS $i^{\prime }$ to actual BFCS $j^{\prime }$, ${I^{\prime }}_{j^{\prime }}$ is the $j^{\prime }$-coordinate representation of origin $I^{\prime }$, $Rot_{i}^{j}$ is the 3-by-3 ATM from the ideal BFCS i to the ideal BFCS j, $I_{j}$ is the j-coordinate representation of the origin I of the ideal BFCS i (see Section 3.2), $Rot_{i}^{i^{\prime }}$ ($Rot_{j}^{j^{\prime }}$) is the 3-by-3 ATM from the ideal BFCS i (j) to the actual BFCS $i^{\prime }$ ($j^{\prime }$), ${J^{\prime }}_{j}$ is the j-coordinate representation of the origin $J^{\prime }$ of the actual BFCS $j^{\prime }$, and $\left(I_{j}+Rot_{i}^{j}{I^{\prime }}_{i}\right)$ is the j-coordinate representation of the origin $I^{\prime }$ of the actual BFCS $i^{\prime }$. The explicit expressions for ${I^{\prime }}_{i}$, ${J^{\prime }}_{j}$, $Rot_{i}^{i^{\prime }}$, and $Rot_{j}^{j^{\prime }}$ are as follows:

(14) $$\{\begin{array}{l} {I^{\prime }}_{i}={\left[\begin{array}{lll} x_{i,ma} & y_{i,ma} & z_{i,ma} \end{array}\right]}^{T}\\ {J^{\prime }}_{j}={\left[\begin{array}{lll} x_{j,ma} & y_{j,ma} & z_{j,ma} \end{array}\right]}^{T}\\ Rot_{i}^{i^{\prime }}=Rot_{y}\left(\theta_{i,ma}\right)Rot_{x}\left(\varphi_{i,ma}\right)Rot_{z}\left(\psi_{i,ma}\right)\\ Rot_{j}^{j^{\prime }}=Rot_{y}\left(\theta_{j,ma}\right)Rot_{x}\left(\varphi_{j,ma}\right)Rot_{z}\left(\psi_{j,ma}\right) \end{array}$$

### 3.4. Actual ABP Determination Model

Since neither the ECF coordinate system nor the FRA coordinate system undergoes thermal deformations, the GPV ${\tilde{L1}}_{\mathrm{FRA},\mathrm{out}}$ (i.e., the ECF-emitted beam defined in the FRA coordinate system in the presence of reflector thermal distortion effects) should have the same form as Equations (4) and (5):

(15) $$\{\begin{array}{l} {\tilde{L1}}_{\mathrm{FRA},\mathrm{out}}=M_{\mathrm{ECF}}^{\mathrm{FRA}}\left(t\right){\tilde{L1}}_{\mathrm{ECF},\mathrm{out}}\\ {\tilde{L1}}_{\mathrm{ECF},\mathrm{out}}={\left[\begin{array}{lllllll} 0 & 0 & 1 & 0 & 0 & 0 & 1 \end{array}\right]}^{T} \end{array}$$

For the explicit expression of $M_{\mathrm{ECF}}^{\mathrm{FRA}}\left(t\right)$, please refer to Equations (5) and (6).

To obtain the GPV ${\tilde{L2}}_{{\mathrm{SSR}}^{\prime },\mathrm{out}}$ representing the beam reflected off the deformed SSR, we can first use the GTM $M_{\mathrm{FRA}}^{{\mathrm{SSR}}^{\prime }}$ to transform the ECF-emitted ${\tilde{L1}}_{\mathrm{FRA},\mathrm{out}}$ defined in the FRA coordinate system into the incident beam ${\tilde{L1}}_{{\mathrm{SSR}}^{\prime },\mathrm{in}}$ defined in the actual SSR coordinate system. Here, ${\tilde{L1}}_{{\mathrm{SSR}}^{\prime },\mathrm{in}}$ can be computed as follows (the calculation of $M_{\mathrm{FRA}}^{{\mathrm{SSR}}^{\prime }}$ can be performed by referring to Equations (13) and (14)):

(16) $$\{\begin{array}{l} {\tilde{L1}}_{{\mathrm{SSR}}^{\prime },\mathrm{in}}=M_{\mathrm{FRA}}^{{\mathrm{SSR}}^{\prime }}{\tilde{L1}}_{\mathrm{FRA},\mathrm{out}}\\ M_{\mathrm{FRA}}^{{\mathrm{SSR}}^{\prime }}=\left[\begin{array}{lll} Rot_{\mathrm{FRA}}^{{\mathrm{SSR}}^{\prime }} & O_{3\times 3} & O_{3\times 1}\\ O_{3\times 3} & Rot_{\mathrm{FRA}}^{{\mathrm{SSR}}^{\prime }} & B_{{\mathrm{SSR}}^{\prime }}\\ O_{1\times 3} & O_{1\times 3} & 1 \end{array}\right] \end{array}$$

where $Rot_{\mathrm{FRA}}^{{\mathrm{SSR}}^{\prime }}$ is the 3-by-3 ATM from the FRA coordinate system to the actual SSR coordinate system and $B_{{\mathrm{SSR}}^{\prime }}$ is the SSR′ coordinate representation of the origin B of the FRA coordinate system. Explicitly, $Rot_{\mathrm{FRA}}^{{\mathrm{SSR}}^{\prime }}$ and $B_{{\mathrm{SSR}}^{\prime }}$ take the following form:

(17) $$\{\begin{array}{l} Rot_{\mathrm{FRA}}^{{\mathrm{SSR}}^{\prime }}=Rot_{\mathrm{SSR}}^{{\mathrm{SSR}}^{\prime }}\left(\varphi_{\mathrm{SSR},ma},\theta_{\mathrm{SSR},ma},\psi_{\mathrm{SSR},ma}\right)Rot_{\mathrm{FRA}}^{\mathrm{SSR}}\\ B_{{\mathrm{SSR}}^{\prime }}=Rot_{\mathrm{SSR}}^{{\mathrm{SSR}}^{\prime }}\left(\varphi_{\mathrm{SSR},ma},\theta_{\mathrm{SSR},ma},\psi_{\mathrm{SSR},ma}\right)\left(-{C^{\prime }}_{\mathrm{SSR}}+B_{\mathrm{SSR}}\right) \end{array}$$

where $Rot_{\mathrm{SSR}}^{{\mathrm{SSR}}^{\prime }}\left(\varphi_{\mathrm{SSR},ma},\theta_{\mathrm{SSR},ma},\psi_{\mathrm{SSR},ma}\right)$ is the 3-by-3 ATM from the ideal SSR coordinate system to the actual SSR coordinate system and ${C^{\prime }}_{\mathrm{SSR}}={\left[\begin{array}{lll} x_{\mathrm{SSR},ma} & y_{\mathrm{SSR},ma} & z_{\mathrm{SSR},ma} \end{array}\right]}^{T}$ is the SSR coordinate representation of the origin $C^{\prime }$ of the actual SSR coordinate system. Obviously, $M_{\mathrm{FRA}}^{{\mathrm{SSR}}^{\prime }}$ is a function of the TDEs of the SSR (i.e., $M_{\mathrm{FRA}}^{{\mathrm{SSR}}^{\prime }}=M_{\mathrm{FRA}}^{{\mathrm{SSR}}^{\prime }}\left(\varphi_{\mathrm{SSR},ma},\;\cdots ,z_{\mathrm{SSR},ma}\right)$).

In a similar manner to that used in Equation (11), ${\tilde{L2}}_{{\mathrm{SSR}}^{\prime },\mathrm{out}}$ can be calculated as

(18) $${\tilde{L2}}_{{\mathrm{SSR}}^{\prime },\mathrm{out}}=F_{{\mathrm{SSR}}^{\prime }}\left(p_{1}^{\prime }\right){\tilde{L1}}_{{\mathrm{SSR}}^{\prime },\mathrm{in}}$$

where $F_{{\mathrm{SSR}}^{\prime }}\left(p_{1}^{\prime }\right)$ is the GRM of the deformed SSR at the POI $p_{1}^{\prime }$. Note that the equation of the SSR (i.e., z=0) is defined in the actual SSR coordinate system when the SSR is thermally deformed, so $F_{{\mathrm{SSR}}^{\prime }}\left(p_{1}^{\prime }\right)$ can be directly computed by using Equations (8)–(10).

From the calculation of ${\tilde{L2}}_{{\mathrm{SSR}}^{\prime },\mathrm{out}}$, as shown above, we can see that the difference between the actual and ideal ABP determinations comes from the GPV transformation between adjacent reflectors’ BFCSs. $M_{{\mathrm{SSR}}^{\prime }}^{{\mathrm{FSR}}^{\prime }}\left(\varphi_{\mathrm{SSR},ma},\;\cdots ,z_{\mathrm{SSR},ma};\varphi_{\mathrm{FSR},ma},\;\cdots ,z_{\mathrm{FSR},ma}\right)$ (the GTM from the actual SSR coordinate system to the actual FSR coordinate system), $M_{{\mathrm{FSR}}^{\prime }}^{{\mathrm{MR}}^{\prime }}\left(\varphi_{\mathrm{FSR},ma},\;\cdots ,z_{\mathrm{FSR},ma};\varphi_{\mathrm{MR},ma},\;\cdots ,z_{\mathrm{MR},ma}\right)$ (the GTM from the actual FSR coordinate system to the actual MR coordinate system), and $M_{{\mathrm{MR}}^{\prime }}^{\mathrm{ICS}}\left(\varphi_{\mathrm{MR},ma},\;\cdots ,z_{\mathrm{MR},ma}\right)$ (the GTM from the actual MR coordinate system to the ICS) can be computed in a similar manner to that used in calculating $M_{\mathrm{FRA}}^{{\mathrm{SSR}}^{\prime }}=M_{\mathrm{FRA}}^{{\mathrm{SSR}}^{\prime }}\left(\varphi_{\mathrm{SSR},ma},\;\cdots ,z_{\mathrm{SSR},ma}\right)$, which will not be repeated here.

According to the above calculations, the actual ABP determination model can be expressed in the following form:

(19) $$\{\begin{array}{l} {\tilde{L}}_{\mathrm{ICS},\mathrm{out}}\left(t\right)=M_{{\mathrm{MR}}^{\prime }}^{\mathrm{ICS}}F_{{\mathrm{MR}}^{\prime }}\left(p_{3}^{\prime }\right)M_{{\mathrm{FSR}}^{\prime }}^{{\mathrm{MR}}^{\prime }}F_{{\mathrm{FSR}}^{\prime }}\left(p_{2}^{\prime }\right)M_{{\mathrm{SSR}}^{\prime }}^{{\mathrm{FSR}}^{\prime }}F_{{\mathrm{SSR}}^{\prime }}\left(p_{1}^{\prime }\right)M_{\mathrm{FRA}}^{{\mathrm{SSR}}^{\prime }}M_{\mathrm{ECF}}^{\mathrm{FRA}}\left(t\right){\tilde{L1}}_{\mathrm{ECF},\mathrm{out}}\\ {\tilde{L1}}_{\mathrm{ECF},\mathrm{out}}={\left[\begin{array}{lllllll} 0 & 0 & 1 & 0 & 0 & 0 & 1 \end{array}\right]}^{T} \end{array}$$

where $F_{{\mathrm{FSR}}^{\prime }}\left(p_{2}^{\prime }\right)$ represents the GRM of the deformed FSR at the POI $p_{2}^{\prime }$ and $F_{{\mathrm{MR}}^{\prime }}\left(p_{3}^{\prime }\right)$ represents the GRM of the deformed MR at the POI $p_{3}^{\prime }$.

### 3.5. Calculation Method for the Thermally Induced BPE

From Section 3.2, Section 3.3 and Section 3.4, we know that the ICS coordinate representation of the ideal pointing of the beam emerging from the MR is $L_{\mathrm{ICS},\mathrm{out}}$ (whose PV component is $l_{\mathrm{ICS},\mathrm{out}}$), while the actual pointing of the emergent beam becomes ${\tilde{L}}_{\mathrm{ICS},\mathrm{out}}$ (whose PV component is ${\tilde{l}}_{\mathrm{ICS},\mathrm{out}}$) by considering the thermal distortion effects of the antenna reflectors. To quantify the impact of the reflector TDEs on the BPE, we define the angular distance between $l_{\mathrm{ICS},\mathrm{out}}$ and ${\tilde{l}}_{\mathrm{ICS},\mathrm{out}}$ as the thermally induced BPE ξ (unit: rad):

(20) $$\xi =\cos^{-1}\left({\tilde{l}}_{\mathrm{ICS},\mathrm{out}}\cdot l_{\mathrm{ICS},\mathrm{out}}\right)=\cos^{-1}\left(\frac{{\tilde{l}}_{\mathrm{ICS},\mathrm{out}}\cdot l_{\mathrm{ICS},\mathrm{out}}}{\left\|{\tilde{l}}_{\mathrm{ICS},\mathrm{out}}\|\times \|l_{\mathrm{ICS},\mathrm{out}}\right\|}\right)\in \left[0,\pi \right]$$

where ${\tilde{l}}_{\mathrm{ICS},\mathrm{out}}\cdot l_{\mathrm{ICS},\mathrm{out}}$ represents the inner product between ${\tilde{l}}_{\mathrm{ICS},\mathrm{out}}$ and $l_{\mathrm{ICS},\mathrm{out}}$ and ‖·‖ represents the modulus of the vector.

Considering that the reflector TDEs may lead to different BPEs within one complete $360^{{}^{\circ}}$ scan cycle of the ECF, we take the root-mean-square (RMS) of the BPEs obtained at different ECF scan azimuths as the overall thermally induced BPE:

(21) $$\xi_{\mathrm{RMSE}}=\sqrt{\overset{N}{\underset{i=1}{\sum }}{\left(\xi_{i}\right)}^{2}/N}$$

where $\xi_{\mathrm{RMSE}}$ is the so-called root-mean-square error (RMSE) of the ABP and N is the number of BPEs obtained at different ECF scan azimuths.

## 4. Simulation Experiments and Discussion

### 4.1. Experimental Design

To verify the overall performance of the proposed ABP determination model and analyze the impact of different reflector TDEs on the BPE, two groups of experiments are presented in this section.

Experiment 1. The proposed ideal ABP determination model and the reflector antenna analysis software (i.e., GRASP) were used to conduct a comparative experiment for ABP determination. The outputs from our MO-based model were compared with the simulation results of the GRASP software to evaluate the ABP determination accuracy of our model. In addition, to verify the advantage of our model in computational efficiency, we also measured the average time for a single ABP spent by our model and the GRASP software. Since the correctness of the BPE analysis is based on the correctness of the validation of the deduced ABP determination models, this experiment also set the basis for the correctness of the beam-pointing error analyses to be presented in Experiment 2.

Experiment 2. The MO-based method for rapid calculation of the thermally induced BPE was used to quantify the independent impact of each TDE on the BPE. Based on these independent impact assessments, the TDEs were ranked according to the degree of impact on the BPE. The purpose of this experiment was to identify the TDEs that had a significant impact on the BPE, thus providing basic guidelines for the on-board thermal distortion compensation scheme design of the examined GEO microwave radiometer.

### 4.2. Experimental Data

The design parameters of the antenna system used in conducting the experiments mentioned above were acquired from the instrument manufacturer and were set as follows:

(1) For the MR, the aperture was $D_{0}=5.0\;m$, the focal length was f=3.996 m, and the offset height was $h_{0}=3.899\;m$.
(2) For the FSR, the aperture was $D_{1}=0.96\;m$, the inter-focal distance was 2c=5.725 m, the major axis length was 2a=4.453 m, and the offset height was $h_{1}=0.682\;m$.
(3) For the SSR, the aperture was $D_{2}=1.4\;m$.
(4) The output of the ECF was set to a standard Gaussian beam, the taper angle was $16.5^{{}^{\circ}}$, the taper was −20 dB, and the initial scan azimuth was $\phi_{q0}=305^{{}^{\circ}}$.

Finally, it should be noted that all experiments in this study were conducted on a Windows 10 computer with an Intel i7-9700K/3.6GHz CPU and 32 GB of memory. The ABP determination model was implemented as C++ code in the Microsoft Visual Studio 2019 programming environment. GRASP software version 10.3 was used.

### 4.3. Experiment 1: Validation of the MO-Based ABP Determination Model

In order to verify the correctness of the MO-based ABP determination model (which we refer to hereafter as the MO-based model) presented in this study, it was necessary to choose an evaluation benchmark. The software GRASP developed by TICRA (http://www.ticra.com/ accessed on 6 July 2021) uses the PO approximation to calculate the 3-D far field antenna patterns at different frequency bands, which is considered the most reliable tool for the analysis of reflector antennas. Therefore, we set the simulation results of GRASP as the reference and employed the angular distance between the ABP from the MO-based model and the reference ABP to evaluate the ABP determination accuracy of the model.

**Figure 5:**
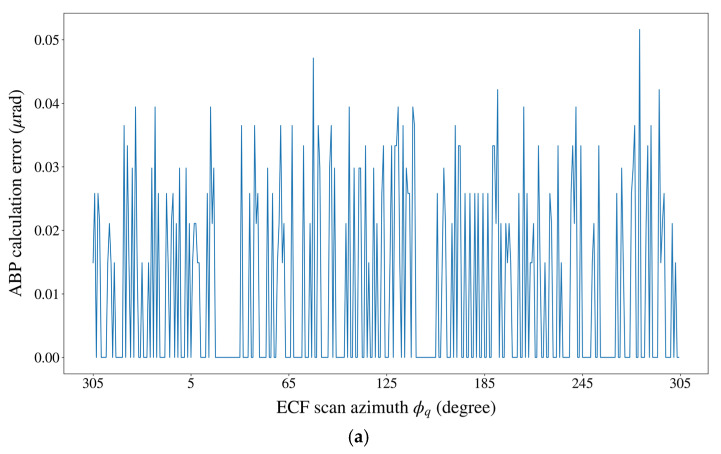

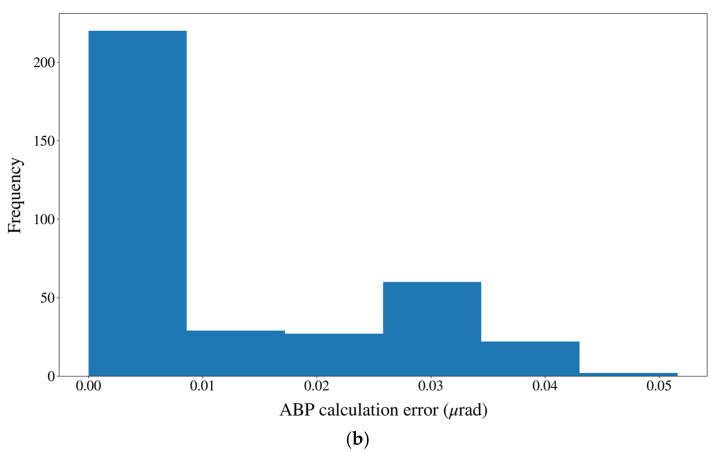
The calculation errors of the MO-based model for ABP determination. (**a**) The variation of the ABP calculation error within one complete 360° scan cycle of the ECF. (**b**) The histogram of the ABP calculation errors.

Due to the limitation of the response time performance of the GRASP software, we took the 54 GHz frequency band (the lowest frequency band to be used) as an example to implement the comparative experiment for ABP determination. To ensure statistical reliability, we used the GRASP software (steps required to execute the GRASP software (e.g., defining the geometrical and electrical objects, selecting the PO method of calculation, and setting the commands for running the analysis of the antenna) can be found in the manual of GRASP [31]) and the MO-based model to calculate the ABP at intervals of $1^{{}^{\circ}}$ within one complete $360^{{}^{\circ}}$ scan cycle of the ECF. Figure 5 shows the calculation errors of the MO-based model for ABP determination. Table 1 gives a statistical summary of the ABP calculation errors presented in Figure 5.

It can be seen from the above experimental results that the MO-based model and the GRASP software generated almost the same results for ABP determination. The ABP calculation errors of the MO-based model showed an irregular fluctuating pattern within the $360^{{}^{\circ}}$ scan cycle of the ECF, which could reach a maximum of $5.16\times 10^{-2}\;\mu \mathrm{rad}$ at the ECF scan azimuth of $280^{{}^{\circ}}$. According to the current system design, the scan azimuth of the ECF ranged from $305^{{}^{\circ}}$ to $55^{{}^{\circ}}$ during scanning of the Earth. Thus, this maximum ABP calculation error would have no direct impact on the geolocation accuracy of the radiometric data. When the ECF scan azimuth was in the range of $305^{{}^{\circ}}$–$55^{{}^{\circ}}$, the maximum ABP calculation error of the MO-based model was $3.94\times 10^{-2}\;\mu \mathrm{rad}$, which was equivalent to a positioning deviation of 1.4 m at the GEO SSP. Compared with the currently achievable SSP spatial resolution at 54 GHz (~60 km), this error could be totally ignored with respect to the NWP operational requirements.

As for time consumption, the MO-based model only took about 0.011 ms on average to complete a single ABP determination. On the contrary, the GRASP software took approximately 50 s to realize one ABP determination (which was about 4,730,000 times that of the MO-based model). This was mainly because the GRASP software consumed extra time in calculating the 3-D far field antenna pattern. In particular, the time for calculating the 3-D antenna pattern by GRASP would increase dramatically with the increase in the beam operating frequency, and it was even impossible for GRASP to achieve one ABP determination within an acceptable time (as the beam operating frequency was increased to 425 GHz (the highest frequency band to be used), the GRASP software still could not complete one ABP determination after 2 h). However, the time consumption of the MO-based model was frequency-independent, which means that the MO-based model would exhibit a more significant advantage in computational efficiency as the beam operating frequency increased.

Finally, it must be pointed out that since the QOF system described in Section 2.1 can allow the electrical boresights of different frequency bands to be coaxially aligned on a common boresight, the experimental results presented herein are the same for different frequency bands, except for the computational time consumed by the GRASP software in calculating the 3-D far field antenna pattern. In other words, while the experimental results were generated for particular operating frequencies, they were representative of any operating frequency to be used for the future GEO microwave radiometer.

In conclusion, the MO-based ABP determination model could achieve a comparable ABP determination accuracy to that of the PO approximation with a significant improvement in computation time. Therefore, the MO-based model could completely replace the PO approximation for real-time ABP determination on the GEO satellite.

### 4.4. Experiment 2: Analysis of the Reflector TDE Impacts on ABP Accuracy

According to Section 3.4, we can see that the thermally induced BPE was related to a total of 18 TDEs ($\varphi_{\mathrm{SSR},ma}$, $\theta_{\mathrm{SSR},ma}$, $\psi_{\mathrm{SSR},ma}$, ⋯, $\varphi_{\mathrm{MR},ma}$, $\theta_{\mathrm{MR},ma}$, $\psi_{\mathrm{MR},ma}$, $x_{\mathrm{SSR},ma}$, $y_{\mathrm{SSR},ma}$, $z_{\mathrm{SSR},ma}$, ⋯, $x_{\mathrm{MR},ma}$, $y_{\mathrm{MR},ma}$, and $z_{\mathrm{MR},ma}$) belonging to 3 reflectors of the examined antenna. These 18 TDEs could be classified into two categories: (1) thermal misalignment errors ($\varphi_{\mathrm{SSR},ma}$, $\theta_{\mathrm{SSR},ma}$, $\psi_{\mathrm{SSR},ma}$, ⋯, $\varphi_{\mathrm{MR},ma}$, $\theta_{\mathrm{MR},ma}$, and $\psi_{\mathrm{MR},ma}$) and (2) thermal translation errors ($x_{\mathrm{SSR},ma}$, $y_{\mathrm{SSR},ma}$, $z_{\mathrm{SSR},ma}$, ⋯, $x_{\mathrm{MR},ma}$, $y_{\mathrm{MR},ma}$, and $z_{\mathrm{MR},ma}$). Because thermal misalignment errors and thermal translation errors have different physical dimensions, it was inappropriate to compare these two kinds of errors horizontally. Accordingly, the impacts of these two kinds of TDEs will be discussed separately in this subsection.

#### 4.4.1. Analysis for Thermal Misalignment Errors

**Figure 6:**
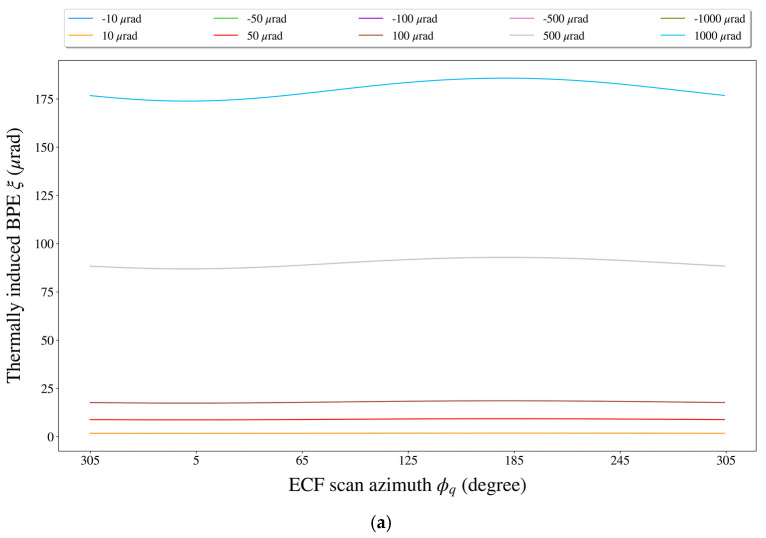

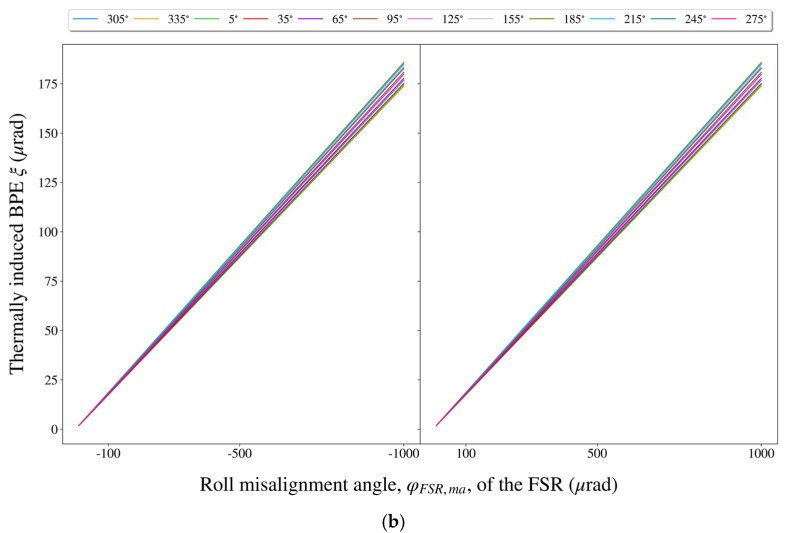
The impact of the roll misalignment angle $\varphi_{\mathrm{FSR},ma}$ of the FSR on the BPE. (**a**) The variation of the BPE with respect to the ECF scan azimuth at some fixed levels of $\varphi_{\mathrm{FSR},ma}$. (**b**) The variation of the BPE with respect to $\varphi_{\mathrm{FSR},ma}$ at some fixed ECF scan azimuths.

**Figure 7:**
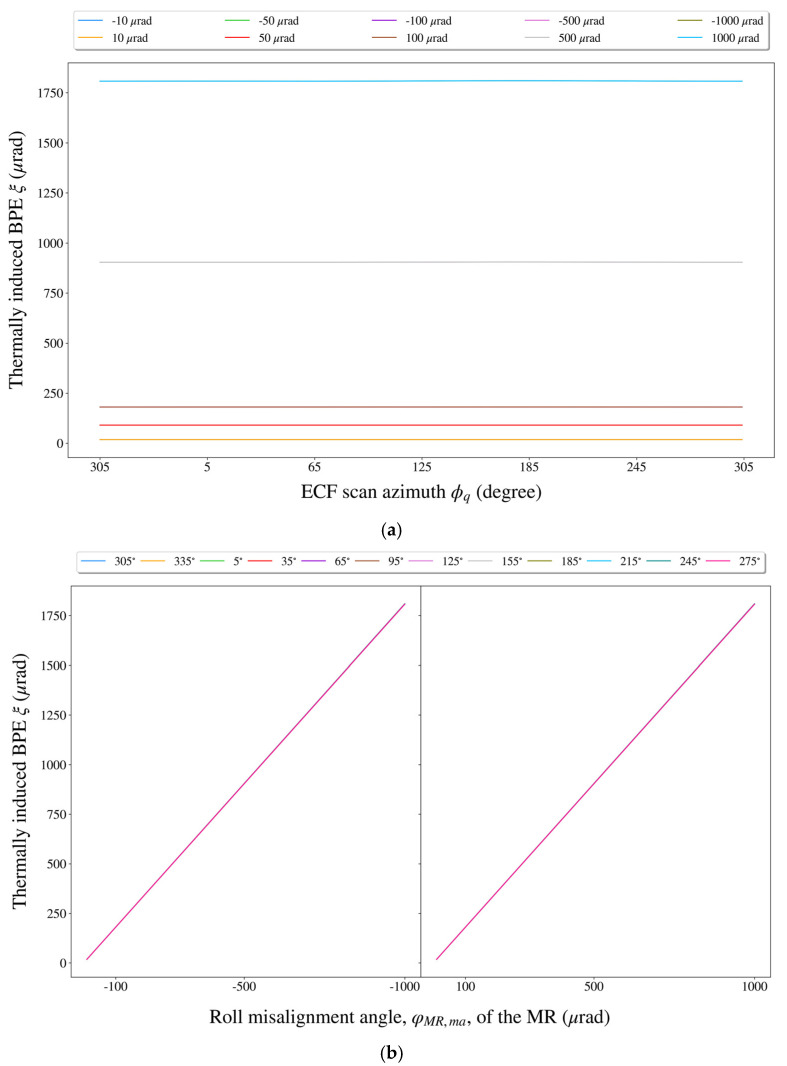
The impact of the roll misalignment angle $\varphi_{\mathrm{MR},ma}$. of the MR on the BPE. (**a**) The variation of the BPE with respect to the ECF scan azimuth at some fixed levels of $\varphi_{\mathrm{MR},ma}$. (**b**) The variation of the BPE with respect to $\varphi_{\mathrm{MR},ma}$ at some fixed ECF scan azimuths.

First, we used the “control variate method” to analyze the BPE caused by each thermal misalignment error (i.e., when analyzing the impact of a certain thermal misalignment error on the BPE, all other TDEs were set to zero). To explore the relationship between the thermally induced BPE, thermal misalignment error, and ECF scan azimuth, the BPE was calculated for every $1^{{}^{\circ}}$ of the ECF scan azimuth as the thermal misalignment error was set to ±10 μrad, ±50 μrad, ±100 μrad, ±500 μrad, and ±1000 μrad (note that counterclockwise rotations are positive while clockwise rotations are negative). Here, we chose some representative experimental results, which are shown in Figure 6 and Figure 7.

(1) It can be seen from Figure 6b that once $\phi_{q}$ was specified, the impact of $\varphi_{\mathrm{FSR},ma}$ on the BPE increased approximately linearly with the increase in $\left|\varphi_{\mathrm{FSR},ma}\right|$.
(2) A careful look at Figure 6a shows that, for a specified level of $\varphi_{\mathrm{FSR},ma}$, the resulting BPE curve showed a significant sinusoidal wave pattern over a scan period of the ECF, with the minimum appearing near $\phi_{q}=0^{{}^{\circ}}$ and the maximum near $\phi_{q}=180^{{}^{\circ}}$. Furthermore, it could be found that the sinusoidal trend in the BPE curve became much more evident as $\varphi_{\mathrm{FSR},ma}$ increased. The reason for this phenomenon was that the rate at which the BPE increased changed with $\phi_{q}$ (manifested as a dispersion of the BPE growth rates at different values of $\phi_{q}$ in Figure 6b). As $\varphi_{\mathrm{FSR},ma}$ varied from ±10 μrad to ±1000 μrad, the dispersion of the BPE growth rates at different $\phi_{q}$ values was widened; thus, the fluctuation of the BPE curve became quite pronounced.
(3) Quantitatively speaking, the fluctuation range (i.e., the difference between the maximum BPE and the minimum BPE) of the BPEs was only ~0.1 μrad with $\varphi_{\mathrm{FSR},ma}=\pm 10\;\mu \mathrm{rad}$; however, when $\varphi_{\mathrm{FSR},ma}=\pm 1000\;\mu \mathrm{rad}$, the fluctuation range of the BPEs could reach ~12 μrad.

Based on the above experimental results, we could draw the following conclusions:

(1) According to Figure 7a, the BPE caused by the roll misalignment angle $\varphi_{\mathrm{MR},ma}$ of the MR was primarily related to $\varphi_{\mathrm{MR},ma}$ itself and did not change with the scan azimuth $\phi_{q}$ of the ECF.
(2) From Figure 7b, once $\phi_{q}$ was specified, the $\varphi_{\mathrm{MR},ma}$-induced BPE increased approximately linearly with the increase in $\left|\varphi_{\mathrm{MR},ma}\right|$.
(3) A careful look at Figure 7a shows that there was not much change in the shape of the BPE curve as $\varphi_{\mathrm{MR},ma}$ varied from ±10 μrad to ±1000 μrad, which was mainly because of the consistency of the BPE growth rates at different values of $\phi_{q}$ (see Figure 7b).
(4) Quantitatively speaking, the fluctuation range of the BPEs was only ~0.02 μrad with $\varphi_{\mathrm{MR},ma}=\pm 10\;\mu \mathrm{rad}$, and when $\varphi_{\mathrm{MR},ma}=\pm 1000\;\mu \mathrm{rad}$, the fluctuation range of the BPEs was still only ~2 μrad.

**Figure 8:**
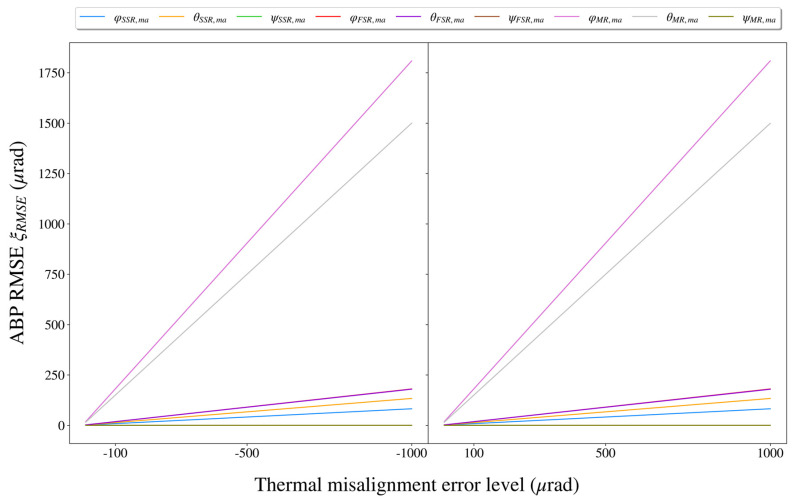
The impacts of different thermal misalignment errors at different levels on the BPE. The impact was measured by the RMSE of the ABPs over a scan period of the ECF.

Based on independent impact assessment for each thermal misalignment error, we could provide impact rankings for these errors. For each thermal misalignment error (which was set to ±10 μrad, ±50 μrad, ±100 μrad, ±500 μrad, and ±1000 μrad previously), the RMSE of the ABPs over a scan period of the ECF was calculated using Equation (21), and the results are shown in Figure 8. Particularly, when the thermal misalignment error level reached the maximum of ±1000 μrad. The maximum impact of each thermal misalignment error on the BPE is shown in Table 2.

Based on the experimental results obtained in this subsection, the following conclusions could be drawn:

(1) Among the thermal misalignment errors, $\psi_{\mathrm{SSR},ma}$, $\psi_{\mathrm{FSR},ma}$, and $\psi_{\mathrm{MR},ma}$ had no impact on the thermally induced BPE. For the remaining thermal misalignment errors, $\varphi_{\mathrm{MR},ma}$ and $\theta_{\mathrm{MR},ma}$ had the highest impacts on the BPE, followed by $\varphi_{\mathrm{FSR},ma}$ and $\theta_{\mathrm{FSR},ma}$, while $\varphi_{\mathrm{SSR},ma}$ and $\theta_{\mathrm{SSR},ma}$ had the lowest impacts. Specifically, the impacts of $\varphi_{\mathrm{MR},ma}$ and $\theta_{\mathrm{MR},ma}$ were by one order of magnitude substantially higher than those of $\varphi_{\mathrm{FSR},ma}$, $\theta_{\mathrm{FSR},ma}$, $\varphi_{\mathrm{SSR},ma}$, and $\theta_{\mathrm{SSR},ma}$ at the same level.
(2) Once the scan azimuth of the ECF (i.e., $\phi_{q}$) was specified, there was an approximately positive linear relationship between the thermal misalignment error and the resulting BPE (i.e., as the thermal misalignment error increased, the resulting BPE tended to increase as well). On the other hand, once the thermal misalignment error level was specified, most thermal misalignment errors (except for $\varphi_{\mathrm{MR},ma}$) led to sinusoidal BPEs over a scan period of the ECF. The fluctuation range of the BPEs was generally in the magnitude of $\sim 10^{-1}\;\mu \mathrm{rad}$ with a small thermal misalignment error; however, as the thermal misalignment error increased, the fluctuation of the BPEs would gradually become pronounced, and the fluctuation range could reach the magnitude of ~10 μrad.

#### 4.4.2. Analysis for Thermal Translation Errors

**Figure 9:**
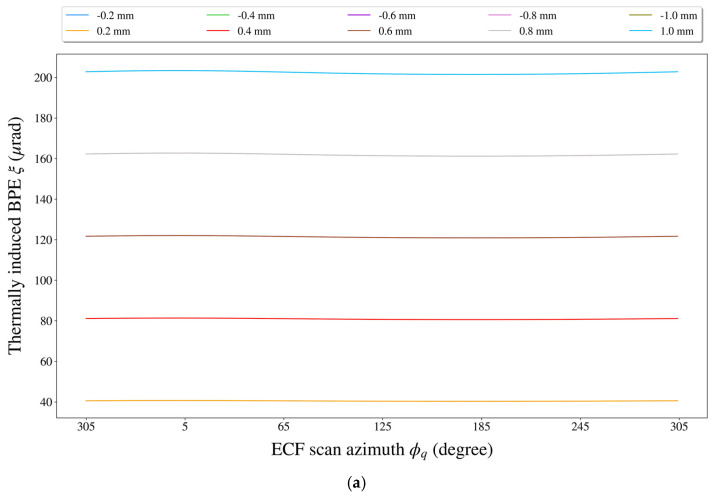

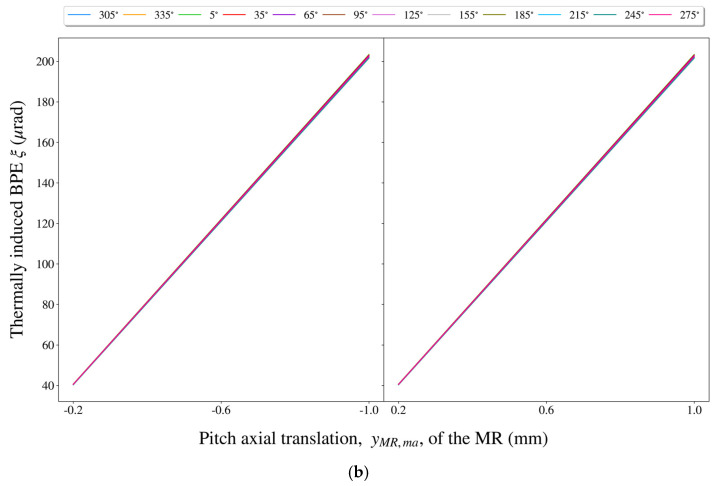
The impact of the pitch axial translation $y_{\mathrm{MR},ma}$. of the MR on the BPE. (**a**) The variation of the BPE with respect to the ECF scan azimuth at some fixed levels of $y_{\mathrm{MR},ma}$. (**b**) The variation of the BPE with respect to $y_{\mathrm{MR},ma}$ at some fixed ECF scan azimuths.

Next, by using the “control variate method” as previously mentioned, we analyzed the independent impact of each thermal translation error on the BPE. To explore the relationship between the thermally induced BPE, thermal translation error, and ECF scan azimuth, the BPE was calculated for every $1^{{}^{\circ}}$ of the ECF scan azimuth as the thermal translation error was set to ±0.2 mm, ±0.4 mm, ±0.6 mm, ±0.8 mm, and ±1.0 mm. As an example, the independent impact of the pitch axial translation $y_{\mathrm{MR},ma}$ of the MR on the BPE was shown in Figure 9.

Based on the above experimental results, similar conclusions could be drawn:

(1) According to Figure 9, the BPE caused by the pitch axial translation $y_{\mathrm{MR},ma}$ of the MR was mainly related to $y_{\mathrm{MR},ma}$ itself and showed very little change with the scan azimuth $\phi_{q}$ of the ECF.
(2) From Figure 9b, once $\phi_{q}$ was specified, the $y_{\mathrm{MR},ma}$-induced BPE increased approximately linearly with the increase in $\left|y_{\mathrm{MR},ma}\right|$.
(3) A careful look at Figure 7a shows that the fluctuation range of the BPEs hardly changed as $y_{\mathrm{MR},ma}$ varied from ±0.2 mm to ±1.0 mm, which was mainly because of the consistency of the BPE growth rates at different values of $\phi_{q}$ (see Figure 9b).
(3) Quantitatively speaking, the fluctuation range of the BPEs was only ~0.4 μrad with $y_{\mathrm{MR},ma}=\pm 0.2\;\mathrm{mm}$, and when $y_{\mathrm{MR},ma}=\pm 1.0\;\mathrm{mm}$, the fluctuation range of the BPEs was still only ~2 μrad.

**Figure 10:**
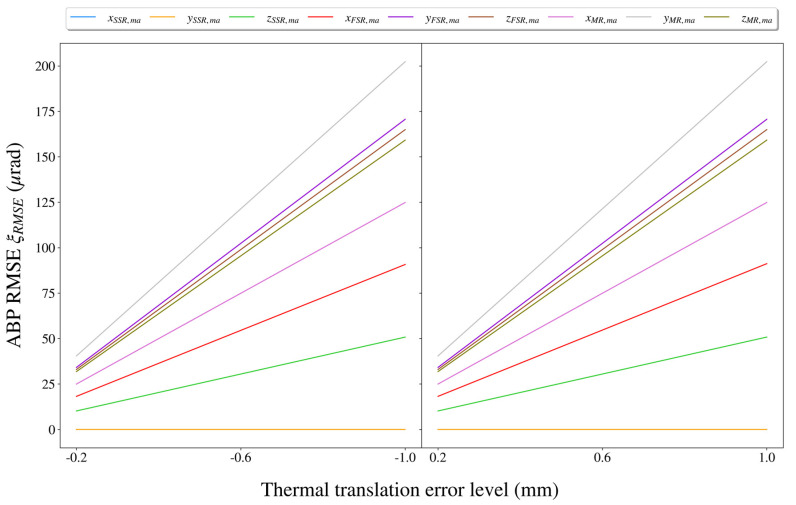
The impacts of different thermal translation errors at different levels on the BPE. The impact was measured by the RMSE of the ABPs over a scan period of the ECF.

Based on independent impact assessment for each thermal translation error, we could provide impact rankings for these errors. For each thermal translation error (which was set to ±0.2 mm, ±0.4 mm, ±0.6 mm, ±0.8 mm, and ±1.0 mm as shown previously), the RMSE of the ABPs over a scan period of the ECF was calculated using Equation (21), and the results are shown in Figure 10. Particularly, when the thermal translation error level reached the maximum of ±1.0 mm, the maximum impact of each thermal translation error on BPE was as shown in Table 3.

Based on the experimental results obtained in this subsection, the following conclusions could be drawn:

(1) Among the thermal translation errors, $x_{\mathrm{SSR},ma}$ and $y_{\mathrm{SSR},ma}$ had no impact on the thermally induced BPE. For the remaining thermal translation errors, their impacts on the BPE had the same order of magnitude. Among them, $y_{\mathrm{MR},ma}$ had the highest impact on the BPE; $y_{\mathrm{FSR},ma}$, $z_{\mathrm{FSR},ma}$, and $z_{\mathrm{MR},ma}$ also had relatively higher impacts; $x_{\mathrm{MR},ma}$ and $x_{\mathrm{FSR},ma}$ had relatively lower impacts; and $z_{\mathrm{SSR},ma}$ had the lowest impact. Specifically, the impact of $y_{\mathrm{MR},ma}$ was about four times larger than that of $z_{\mathrm{SSR},ma}$.
(2) Once the scan azimuth of the ECF (i.e., $\phi_{q}$) was specified, there was an approximately positive linear relationship between the thermal translation error and the resulting BPE. On the other hand, once the thermal translation error level was specified, most thermal translation errors (except for $y_{\mathrm{FSR},ma}$, $y_{\mathrm{MR},ma}$, and $z_{\mathrm{MR},ma}$) led to sinusoidal BPEs over a scan period of the ECF. The fluctuation range of the BPEs was generally 1~2 μrad with a small thermal translation error; however, as the thermal translation error increased, the fluctuation of the BPEs would gradually become pronounced, and the fluctuation range could reach a maximum of 10 μrad.

## 5. Conclusions

In this study, the rapid ABP determination and thermally induced BPE analysis method in support of the development of the onboard INR system needed by the GEO mechanical scanning microwave radiometer were studied using the example offset Cassegrain antenna with a planar folding mirror proposed for China’s future GEO microwave radiometer. The main contributions of this study are as follows.

First, we adapted the traditional MO method for modeling the equivalent optical path of the microwave antennas with complicated configurations and deduced the ideal and actual ABP determination models for the examined GEO antenna. Compared with the classical electromechanical coupling principle, the MO-based ABP determination model avoids the complex calculations such as finite element thermoanalysis and PO approximation, so it is advantageous in terms of speed performance and technical implementability. Thus, it is more suitable for the in-orbit INR technique (for future development) needed by the GEO microwave radiometer. When INR is turned on, the deduced ideal ABP determination model can be used in the 2-D beam scan planning and the direct geolocation of the acquired radiometric data. To verify the overall performance of the MO-based ABP determination model, the comparative experiment for ABP determination was conducted by setting the GRASP 10.3 software as the evaluation benchmark. The experimental results show that the MO-based ABP determination model yielded a significant speed improvement (~4,730,000 times at 54 GHz) relative to the GRASP software, and the maximum ABP calculation error of the MO-based model was only $5.16\times 10^{-2}\;\mu \mathrm{rad}$ within the $360^{{}^{\circ}}$ scan cycle of the ECF, which can be completely ignored with respect to the NWP operational requirements.

Another contribution of this study was to propose an MO-based method for fast calculation of the thermally induced BPE, which established a direct connection between the reflector TDEs and the resulting BPE. With the aid of this MO-based BPE computing method, the impacts of the reflector TDEs on the BPE were quantified on a case-by-case basis, and those TDEs that had a significant impact on the BPE were thus identified. The experimental results provided general conclusions for the thermal distortion compensation scheme design of the classical offset Cassegrain reflector antenna with a planar folding mirror as follows:

(1) The misalignment angles of the yaw axis (focal axis) of the MR and FSR had no impact on the thermally induced BPE, and the misalignment angle of the yaw axis (surface normal) and the roll and pitch axial translation of the SSR (i.e., the planar folding mirror) also had no impact on the BPE.
(2) For thermal misalignment errors, the roll and pitch misalignment angles of the MR had the greatest impacts on the BPE, followed by the thermal misalignment errors of the FSR, and the thermal misalignment errors of the SSR had the lowest impact. The impacts of MR’s thermal misalignment errors were by one order of magnitude substantially higher than those of the thermal misalignment errors of the FSR and SSR.
(3) For the thermal translation errors, their impacts on the BPE had the same order of magnitude. Among them, the pitch axial translation of the MR had the highest impact on the BPE, while the yaw axial translation of the SSR had the lowest impact. The impact of the former was about four times greater than that of the latter. Therefore, to ensure the ABP accuracy required by the GEO microwave radiometer, stringent measures must be adopted to minimize the thermal distortions of the MR.
(4) For any TDE examined in this study, once the scan azimuth of the ECF was specified, there was an approximately positive linear relationship between this TDE and the resulting BPE. On the other hand, once the TDE level was specified, most TDEs led to sinusoidal BPEs over a scan period of the ECF. The greater the TDE was, the more significant the sinusoidal trend in the resulting BPEs. When 13 TDEs existed simultaneously, the maximum fluctuation range of the BPEs was ~62 μrad during scanning of the Earth (i.e., the scan azimuth of the ECF ranged from $305^{{}^{\circ}}$ to $55^{{}^{\circ}}$). This means that it is still feasible to use satellite attitude maneuvering to compensate for the thermally induced BPEs within the current NWP operational requirements.

It should be noted that although the current actual ABP determination model (which is a rigorous model) can exactly compute the actual antenna beam pointing given the true thermal distortion parameters, it cannot be taken directly for the inverse calculation of the antenna thermal distortion parameters due to the problem of over-parameterization. Based on the above-mentioned conclusions, the actual ABP determination model can be further simplified in future research so as to be used to estimate the equivalent thermal distortion parameters at times of interest.

Finally, it should be pointed out that the conclusions presented herein have a certain degree of universality for the three-reflector antenna that utilizes the offset Cassegrain antenna as the main body; and the method for deducing ABP determination models and analyzing the thermal distortion effects on BPE is a general method, which can also benefit the ABP determination and BPE analysis of other antenna configurations to a certain extent.

## Figures and Tables

**Table 1:** The statistical summary of the ABP calculation errors of the MO-based model.

Statistical Category	Statistical Value
Maximum error (μrad)	$5.16\times 10^{-2}$
Mean error (μrad)	$1.02\times 10^{-2}$
RMSE (μrad)	$1.72\times 10^{-2}$

**Table 2:** The maximum impact of each thermal misalignment error on the BPE at the level of ±1000 μrad.

Thermal Misalignment Error	$\varphi_{\mathrm{SSR},ma}$	$\theta_{\mathrm{SSR},ma}$	$\psi_{\mathrm{SSR},ma}$	$\varphi_{\mathrm{FSR},ma}$	$\theta_{\mathrm{FSR},ma}$	$\psi_{\mathrm{FSR},ma}$	$\varphi_{\mathrm{MR},ma}$	$\theta_{\mathrm{MR},ma}$	$\psi_{\mathrm{MR},ma}$
**RMSE of ABPs** (μrad)	81.8	133.2	0.0	180.1	179.2	0.0	1808.7	1499.5	0.0

**Table 3:** The maximum impact of each thermal translation error on the BPE at the level of ±1.0 mm.

Thermal Translation Error	$x_{\mathrm{SSR},ma}$	$y_{\mathrm{SSR},ma}$	$z_{\mathrm{SSR},ma}$	$x_{\mathrm{FSR},ma}$	$y_{\mathrm{FSR},ma}$	$z_{\mathrm{FSR},ma}$	$x_{\mathrm{MR},ma}$	$y_{\mathrm{MR},ma}$	$z_{\mathrm{MR},ma}$
**RMSE of ABPs** (μrad)	0.0	0.0	50.8	91.2	170.6	164.9	124.9	202.3	159.2
